# The impact of the elimination diet on growth and nutrient intake in children with food protein induced gastrointestinal allergies

**DOI:** 10.1186/s13601-016-0115-x

**Published:** 2016-07-14

**Authors:** Rosan Meyer, Claire De Koker, Robert Dziubak, Heather Godwin, Gloria Dominguez-Ortega, Adriana Chebar Lozinsky, Ana-Kristina Skrapac, Yara Gholmie, Kate Reeve, Neil Shah

**Affiliations:** Gastroenterology Department, Great Ormond Street Hospital for Children NHS foundation Trust, London, UK; Department of Nutrition and Dietetics, Chelsea and Westminster Hospital NHS Foundation Trust, London, UK; Niño Jesús University Children Hospital, Madrid, Spain; Paediatric Gastroenterology, Universidade Federal de Sao Paulo (UNIFESP), São Paulo, Brazil; Department of Nutrition and Food Sciences, Faculty of Agricultural and Food Sciences, American University of Beirut, Beirut, Lebanon; Institute of Child Health, University College, London, UK

**Keywords:** Growth, Anthropometric measures, Non-IgE mediated allergy, Malnutrition, Nutrients

## Abstract

**Background:**

Non immunoglobulin E (IgE) mediated allergies affecting the gastrointestinal tract require an elimination diet to aid diagnosis. The elimination diet may entail multiple food eliminations that contribute significantly to macro- and micro-nutrient intake which are essential for normal growth and development. Previous studies have indicated growth faltering in children with IgE-mediated allergy, but limited data is available on those with delayed type allergies. We therefore performed a study to establish the impact on growth before and after commencing an elimination diets in children with food protein induced non-IgE mediated gastrointestinal allergies.

**Methods:**

A prospective, observational study was performed at the tertiary gastroenterology department. Children aged 4 weeks–16 years without non-allergic co-morbidities who were required to follow an elimination diet for suspected food protein induced gastrointestinal allergies were included. Growth parameters pre-elimination were taken from clinical notes and post-elimination measurements (weight and height) were taken a minimum of 4 weeks after the elimination diet. A 3-day estimated food diary was recorded a minimum of 4 weeks after initiating the elimination diet, including also any hypoallergenic formulas or over the counter milk alternatives that were consumed.

**Results:**

We recruited 130 children: 89 (68.5 %) boys and a median age of 23.3 months [IQR 9.4–69.2]. Almost all children (94.8 %) in this study eliminated CM from their diet and average contribution of energy in the form of protein was 13.8 % (SD 3.9), 51.2 % (SD 7.5) from carbohydrates and 35 % (SD 7.5) from fat. In our cohort 9 and 2.8 % were stunted and wasted respectively. There was a statistically significant improvement in weight-for-age (Wtage) after the 4 week elimination diet. The elimination diet itself did not improve any of the growth parameters, but achieving energy and protein intake improved Wtage and WtHt respectively, vitamin and/or mineral supplements and hypoallergenic formulas were positively associated with WtHt and Wtage.

**Conclusion:**

With appropriate dietary advice, including optimal energy and protein intake, hypoallergenic formulas and vitamins and mineral supplementation, growth parameters increased from before to after dietary elimination. These factors were positively associated with growth, irrespective of the type of elimination diet and the numbers of foods eliminated.

## Background

It is thought that between 2.2 and 5.5 % of young children in the United Kingdom (UK) suffer from a proven food allergy [1]. However, this data is mainly based on immunoglobulin E (IgE) mediated allergies, with no known population prevalence data for non-IgE mediated food allergies. Non-IgE mediated food allergies include food protein induced gastrointestinal allergies such as proctocolitis, enterocolitis, eosinophilic gastrointestinal disorders, food protein induced enterocolitis syndrome and enteropathy [2, 3]. The pathophysiology and the diagnostic tests differ between IgE and non-IgE mediated allergies, in that the latter requires an elimination diet followed by the reintroduction of allergens to confirm the diagnosis whereas IgE mediated allergies have the benefit of both skin prick or specific IgE tests [4, 5]. The approach to elimination diets vary amongst allergy centres, with some preferring to eliminate all common allergens initially followed by single reintroduction, whereas others commence children on single food eliminations and increase the number of foods eliminated until symptom resolution occurs [6]. Whatever method is used, it can take several months in children with multiple non-IgE mediated food allergies to identify the correct offending allergen(s) through dietary elimination, which may impact on growth.

The most common foods involved in non-IgE mediated allergies affecting the gastrointestinal tract include: cow’s milk (CM), hen’s egg, soya bean and wheat [3, 6, 7]. Many of these foods, in particular CM, contribute significantly to macro- and micronutrient intake which are essential for normal growth and development, especially during early childhood [8]. Isolauri et al. [9] highlighted poor growth, in particular stunting, as a problem in children on a CM exclusion diet for IgE-mediated allergy. More recently, Flammarion et al. [10] also published growth data on children with IgE-mediated allergy and linked the number of foods excluded to a low weight- and height-for-age (Wtage and Htage) z-score. To date, only one study has been published in a non-IgE mediated population with gastrointestinal symptoms, which found a very high percentage of both wasting [low weight-for-height (WtHt)] and stunting (low Htage) in children [11]. However, this study only reviewed growth in CM allergy and did not focus on other food eliminations; furthermore the growth was not linked to actual dietary intake. Thus, limited information exists on growth in children on elimination diets with food protein induced non-IgE mediated gastrointestinal allergies. We therefore set out to establish the impact on growth before and after an elimination diet in children with non-IgE mediated food allergies affecting the gastrointestinal tract and assessed factors that contributed towards growth.

## Methods

### Subjects

A prospective, observational study was performed at the tertiary gastroenterology department, from Great Ormond Street Hospital for Children NHS Foundation Trust, London, UK. Parents of children aged 4 weeks–16 years without non-allergic co-morbidities (i.e. cerebral palsy, cardiac disorders) who were required to follow an elimination diet for the diagnosis of suspected non-IgE mediated gastrointestinal food allergies, were approached to take part in the study. Inclusion in the study occurred if after 4 weeks of following the elimination diet, there was an improvement in their gastrointestinal symptoms. This was measured by a repeated likert scale gastro-intestinal symptom questionnaire that has previously been published [12].

### Anthropometry

Pre-elimination weight and height measurements were taken from the referral letters, clinical notes and health records, as parents only came in once during the study for assessment, which was after the elimination diet was shown to lead to symptom improvement. At the research appointment, a minimum of 4 weeks after the elimination diet was commenced, weight and height measurements were repeated.

Weight was measured using a SECA (Hamburg, Germany) portable electronic baby (<10 kg), or SECA (Hamburg, Germany) sitting (>10 kg) scales, calibrated as per hospital protocol. Length was measured using a portable recumbent length meter in children under 2 years of age, and a fixed standing height meter in older children (rounded off to the nearest 0.1 decimal). All growth measurements were converted to z-scores using the WHO Anthro (birth—5 years) and AnthroPlus Software (>5–18 years). We assessed the z-scores for Wtage, Htage and WtHt and for children ≤5 years of age and for children >5 years, body mass index (BMI) replaced WtHt in the AnthroPlus Software. We compared the z-scores before and after the elimination diet, where available and assessed the number of children that were stunted (Htage ≤2 z-scores), wasted (WtHt less than or equal to −2 z-score) or overweight (>2 z-score) as defined by the World Health Organisation (WHO) after a minimum of 4 week dietary elimination period [13].

### Dietary intake analysis

The parents of all children in this study received dietetic advice with standard diet sheets published by the Food Allergy Specialist Group of the British Dietetic Association at the time of having to embark on the elimination diet. This advice included not only how to avoid allergenic foods, but also individualised information on a suitable hypoallergenic formula (HF)/over the counter milk and supplementation if required as per dietetic assessment. A 3-day estimated food diary (2 week days and 1 weekend day) was recorded a minimum of 4 weeks after initiating the elimination diet. Carers were given detailed instructions on how to complete the diary as accurately as possible, including a portion size guide and a sample menu. HF consumption (including type and volume) and over-the-counter milk alternatives for older children were also documented. Food diaries were discussed with parents and any unclear entries were clarified by the researcher if possible, either at the time of research appointment or by means of telephone communication.

Nutritional intake data was assessed using Dietplan 6 (Forestfield Software Limited, UK). Any foods, in particular specialist foods free from allergens, as well as HF not available on the software database were manually added by the researcher, and product information was obtained from the manufacturer where necessary.

Dietary intake for energy and protein were compared to the UK Dietary Reference Values using the reference nutrient intake (RNI) for protein and estimated average requirements (EARs) for energy [14]. Insufficient intake for protein was defined as an intake <100 % of the lower reference nutrient intake (LRNI—meeting nutrient requirements for 2.5 % of population), sufficient intake was between the LRNI and <200 % of the RNI and excessive intake >200 % of the RNI [14].

For energy intake, the RNI is not used because it represents an excess energy intake for the majority of the population, as highlighted by the Scientific Advisory Committee on Nutrition in the United Kingdom (SACN) [14]. Instead the EAR were used and children consuming below 67 % were classified as low energy intake, between 67 % EAR and 110 % as sufficient intake and excessive intake in this study was arbitrary based on 110 % of the EAR [15].

### Statistical analysis

Statistical analysis was performed using IBM SPSS Statistics for Windows, version 22 (Armonk, NY, USA). Continuous variables are presented as means with standard deviations or medians with interquartile ranges where appropriate. Categorical variables are presented as frequencies Bonferroni correction was used in univariable analysis. Paired-samples t test was used to compare growth parameters before and after elimination diet and Mann–Whitney U Test was used to compare z-scores between groups of children achieving/not achieving energy intake requirements. Spearman’s test was used to check correlation between percentage energy intake from fat, carbohydrates and proteins, and z-scores. Multivariable regression analysis was used to ascertain the association between anthropometrical parameters and the following parameters: macronutrients (i.e. protein, carbohydrate and fat) and vitamin and/or mineral supplementation, consumption of HF and over-the-counter milk alternatives (i.e. oat, rice, coconut or nut milks), food elimination (i.e. CM, egg, wheat, soya) and number of foods eliminated, time between pre- and post-intervention and gastrointestinal symptoms (i.e. diarrhoea, vomiting, feeding difficulties). We accounted for age and gender in the regression model and factors were only included in the regression analysis based on the outcome of univariate analysis. All tests were two-tailed and significance level was set to 0.05.

## Results

### Subjects

**Fig. 1:**
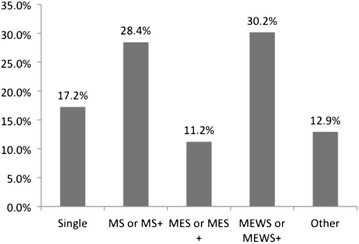
Combinations of food elimination diets. *MS* milk and soya, *MES* milk, egg and soya, *MEWS* milk, egg, wheat and soya, ^+^random additional foods to the list

We recruited 131 children in the study and 130 had available growth parameters. Data was obtained from 89 (68.5 %) boys and 41 (31.5 %) girls with a median age of 23.3 months [IQR 9.4–69.2]. In this study 10.8 % were on a HF only, 17.2 % avoided one food, 30.2 % two foods, 15.5 % three foods and 37.1 % eliminated ≥4 foods. Almost all children (94.8 %) in this study eliminated CM from their diet, 74 % also soya with 45.7 and 44.8 % also avoiding egg and wheat respectively. The most frequent combination (30.2 %) of foods eliminated was CM, soya, egg and wheat (with or without other foods) (Fig. 1).

### Anthropometrics

The mean Wtage, Htage, WtHt (<5 years of age) and BMI z-scores (>5 years of age) for children after a minimum of 4 weeks elimination was 0.044, −0.186, 0.296 and 0.042. Differences in z-scores before and after intervention were calculated where both measurements were available and are presented in Table 1. There was a statistically significant increase in Wtage z-score (p = 0.003) before and after dietary elimination. Although there was an improvement in Htage z-score from −0.155 to −0.122 this was not statistically significant. After following the elimination diet for 4 weeks, 11/130 (9 %) of children were stunted and 2/90 (2.2 %) children <5 years of age were wasted (z-score less than −2) and 2/40 (5 %) >5 years had a BMI less than −2 z-score. Conversely 2/90 (2.2 %) and 4/40 (10 %) children <5 and >5 years of age were overweight respectively with a WtHt or BMI z-score >2.

**Table 1:** Differences in z-scores for measurements before and after the elimination diet

	N	Before		After		Difference	
		Mean	SD	Mean	SD	Mean	p value
Wtage	88	−0.203	1.147	0.008	1.009	0.211	0.003*
Htage	84	−0.155	1.268	−0.122	1.168	0.033	0.688
WtHt (≤5 years)	56	−0.023	1.246	0.260	1.088	0.282	0.039
BMI (>5 years)	27	−0.034	1.094	−0.051	1.112	−0.017	0.824

### Macronutrient intake

**Fig. 2:**
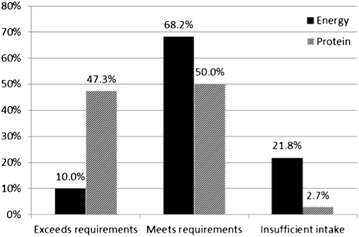
Percentage of patients with insufficient intake, meeting their requirements or excessive intake

Of the 131 patients recruited, 123 had completed food diaries, but only 110 food diaries were included in the data analysis due to some infants being breast fed (difficult to estimate intake on an individual basis) or due to inadequate information on foods or portion sizes. According to our definitions 68.2 and 50.0 % of children met their requirements for energy and protein respectively (Fig. 2). Although not many exceeded their intake for energy (10.0 %), 47.3 % consumed ≥200 % of the RNI for protein. In addition 21.8 % consumed less than the recommended EAR for energy (Fig. 2). In this study, the average contribution of energy in the form of protein was 13.8 % (SD 3.9), 51.2 % (SD 7.5) from carbohydrates and 35 % (SD 7.5) from fat.

### Association between growth parameters and nutritional intake

In this study, 21.8 % did not achieve their EAR for energy versus only 2.7 % not achieving requirements for protein. In fact, 47.3 % exceeded the protein requirements for their age (Fig. 2). We assessed if there were differences in z-scores for all growth parameters between those who achieved and did not achieve sufficient energy intake and did not find any statistical significant association between these (Table 2). This analysis was not performed for protein due to the low number not achieving the protein requirements.

**Table 2:** Association between energy intake and growth parameters expressed as z-scores

Standard	Insufficient energy intake	Meet or exceeds energy requirements	p value
WtHt	−0.01	0.42	0.82
Wtage	−0.33	0.33	0.07
Htage	−0.35	−0.09	0.18

Multivariate regression analysis was performed to assess factors that impacted on the change in growth measurements before and after the elimination diet (Table 3). Table 3 presents these findings, however in summary: those receiving vitamin and/or mineral supplements on average had a bigger increase in WtHt z-score and similarly children that received a higher percentage of energy from protein had on average a bigger rise in WtHt z-scores from before to after the elimination diet. Those achieving EARs for energy had a bigger increase in Wtage z-scores from before to after the elimination diet. Children that received a higher percentage of energy from carbohydrates had on average lower post elimination Htage z-scores.

**Table 3:** Regression analysis on the association of different factors on growth parameters

Variable	Difference WtHt^a^	Post elim. WtHt	Difference in Wtage^a^	Post elim. Wtage	Post elim. Htage
(Constant)	−1.005	0.592	−0.211	0.94	1.355
Pre elimination z score	−0.272**	0.475***	−0.082*	0.702***	0.654***
Hypoallergenic formula			0.234**	0.458***	
Vitamin/mineral supplements	0.685**				
Energy from protein (%)	0.083**				
Achieving energy EAR			0.197*		
Energy from carbohydrate (%)					−0.026**
Over-the-counter milk		−0.510*			
R^2^ (%)	43.4	40.0	18.4	68.9	67.4

* p < 0.05; ** p < 0.01; *** p < 0.001

^a^The difference before and after commencing the elimination diet

The presence of a HF impacted positively on both difference in Wtage z-scores and on post elimination Wtage z-scores, conversely over-the-counter alternative milks negatively impacted on the post elimination WtHt z-scores. Children with higher previous WtHt z-score and Wtage had on average lower change in WtHt and Wtage z-scores respectively during the elimination period. Children with higher previous WtHt, Wtage and Htage z-scores had on average higher post elimination WtHt, Wtage and Htage z-scores. The following variables were not significantly associated with growth in any of the statistical models: gastrointestinal symptoms, foods excluded (milk, wheat, soya, egg) and number of foods excluded.

## Discussion

This study set out to establish growth before and after an elimination diet for presumed non-IgE mediated gastrointestinal food allergies and assessed the impact of macro and micronutrients on these parameters. To the knowledge of the authors this is the first such study focusing on the whole spectrum of non-IgE mediated gastrointestinal allergies and the association between dietary elimination on growth. This study indicated there was an improvement in WtHt and Wtage on the elimination diet, but we found that the elimination diet itself (i.e. CM, soya, egg, wheat) and the number of foods eliminated did not have a positive impact on growth over a 4 week period in our population. However the presence of a HF in addition to achieving energy requirements and percentage protein intake improved the Wtage and WtHt z-score.

In this study stunting was present in 9 % of children after a 4 week elimination diet, but only a very small number of children were wasted (2.2 and 5 %). In the general population the WHO estimates stunting in developed countries to be around 6 % [16]. Flammarion et al. [10] found in an IgE-mediated cohort that 12.1 % of children were stunted and 9.8 % wasted if ≥3 foods were eliminated. Another study performed in Brazil on a non-IgE mediated CM allergic cohort, found much higher levels of stunting and wasting at 23.9 and 8.8 % respectively [11]. The differences in results are most likely related to different populations and also the fact that all of the patients included in our study received individualised dietetic advice using standard diet sheets. Previously published work by Meyer et al. [17] on the nutritional status of children with IgE and non-IgE mediated allergy under dietetic care in the UK have found that 11.9 and 3.7 % were stunted and wasted respectively and that the number of foods eliminated only made an impact on Wtage but not WtHt or Htage. What our current study reinforces, is that in a non-IgE mediated gastrointestinal allergy population on an elimination diet, a significant number of children with this allergy will be stunted irrespective of dietary advice including a suitable HF and vitamin and mineral supplementation [9, 11, 17].

In this study there was an overall improvement in Htage, WtHt, Wtage but only the latter was statistically significant. As there was on average a minimum of 4 weeks between commencing the elimination diet and the research review, there was most probably insufficient time for significant height growth to occur. The average age of our population was around 2 years of age and Himes [18] suggest a minimum time of 52 days in this age group to detect significant changes in height growth. There is concern about short stature in food allergic children, in spite of optimal dietary elimination, which has been highlighted by Isolauri et al. [9] and Meyer et al. [17]. Future studies should aim to review height growth following an elimination diet over a longer period of time to establish the impact of the dietary elimination.

The improvement in WtHt and Wtage found in this study was not associated with the elimination diet itself or the number of foods eliminated. Instead we found that achieving the EAR for energy and the percentage of energy coming from protein (in this study 13.8 %) had a positive impact on Wtage and WtHt. Although it makes sense that better growth is achieved in children that receive their energy requirements, in our study 21.8 % did not achieve the EAR for energy, but seemed to grow well. This is in line with the findings of Flammarion et al. [10] who found that 24 % of children with and 23 % without food allergy also did not achieve their energy requirements, without an apparent impact on their growth. This may be associated with inaccuracies in dietary intake reporting and assessment, however from a clinical perspective our findings still indicate that growth can be improved if EAR for energy is achieved. In contrast to the intake of energy, 47.3 % exceeded their RNI for protein. This is not a novel finding in both food allergic children as well as general paediatric population. Flammarion et al. [10] found in their cohort that the majority of children consumed more than their RNI for protein. The National Diet and Nutrition Survey [19] from the UK also found that in the majority of healthy children, protein contributed around 15 % of energy. Similarly, the European Survey by Lambert et al. [20] established that energy from protein in children from a variety of European countries ranged from 11 to 16.6 %. What is interesting from our study is that protein was positively associated with and improvement in WtHt z-score. The importance of additional protein in catch-up growth has been highlighted by the WHO/FAO/UNO guidelines on protein requirements in 2007 as well as Golden in 2009 [21, 22]. It is thought that up to 15 % of protein may be required in severe stunting [23, 24]. The findings of our study, contribute important information to future dietary management of children with non-IgE mediated food allergies, indicating that a higher level of energy from protein may be required to achieve catch-up in height growth.

In addition to the positive impact of energy and protein, we have also found that the presence of a HF positively impacted on Wtage z-score but not Htage z-score, which is most probably related to there being insufficient time to see changes in height as highlighted above. Conversely, the presence of an over-the- counter milk alternative, negatively impacted on the post elimination WtHt z-score. Over-the-counter milk alternatives (oat milk, rice milk, nut milks) are particularly low in protein and provide on average between 0.1 and 1 g/100 ml of protein, whereas formulas provide 1.6–1.9 g/100 ml of protein (<1 year of age). We hypothesise that the negative impact of these alternative milks is mainly related to the low protein content, as percentage energy from protein has been associated with improved growth. Our group has also recently published the impact of a HF versus over-the-counter alternative on micronutrient intake. We found that micronutrient intake is positively affected by the presence of a HF [25]. This may also affect growth, as in this study the presence of a vitamin/mineral supplement positively impacted on WtHt of these children. We have not been able to isolate specific vitamins or minerals involved, however future studies should assess the impact of vitamin D, zinc, iron and other essential vitamins and minerals on growth.

The limitations of the study include the lack of a control group, which would have enabled a comparison between dietary intake and growth in an allergic and non-allergic cohort. In addition, having a 3 day food diet before and after the elimination diet would have also been beneficial in establishing the impact of nutrient intake on growth. In this study, children were only enrolled after symptom improvement was achieved, which was a major entry criteria for the study, therefore parents were only invited for a growth review after 4 weeks on the elimination diet. This meant that some came a day or two after the 4 week symptom assessment or a week after this. Although this introduces some variation in timing of growth assessment, we believe that this variation in timing would not impact significantly on our growth data. Another limitation of the study is that we did not manage to obtain full growth measurements (weight and height) before and after the elimination diet in all subjects, however we did collect sufficient data to show a significant trend. It would have also been beneficial to have repeated height and weight parameters again 3 months after the elimination diet was commenced to assess the impact on Htage, however this was not possibly with the resources available for the study. In addition, the accuracy of the 3-day food diary needs to be taken into account when interpreting the results. The problems related to accuracy of dietary intake methods have been highlighted by many studies. In our study, we decided on a 3-day semi quantitative food diary, as Lanigan et al. [26] did not find that a weighed record provided a significant benefit over a semi-quantitative diary. In addition a 3-day food diary was chosen instead of a 7 day diary, to reduce the fatigue effect of recording dietary intake for such a long. However, future studies assessing dietary intake my benefit from adding a second dietary intake method to ensure that recorded dietary intake is accurate.

## Conclusion

This study has shown that nutritional management of children with non-IgE mediated gastrointestinal food allergies, significantly impacts on growth. In this study patients had improved growth parameters following dietary elimination. This positive impact was related to energy and protein intake, the use of a HF and vitamin and/or mineral supplementation, irrespective of the type of elimination diet and the numbers of foods eliminated. Future studies should aim to recruit a control group to establish whether these findings are unique to children with this type of allergy and assess the impact of the elimination diet over a longer period.

## Abbreviations

CM: cow’s milk
EAR: estimated average requirements
IgE: immunoglobulin E
Htage: height-for-age
HF: hypoallergenic formula
NHS: National Health Service
OFC: occipito-frontal circumference
RNI: recommended nutrient intake
SACN: Scientific Advisory Committee on Nutrition in the United Kingdom
UK: United Kingdom
WHO: World Health Organisation
Wtage: weight-for-age
WtHt: weight-for-height
